# Lack of increases in methylation at three CpG-rich genomic loci in non-mitotic adult tissues during aging

**DOI:** 10.1186/1471-2350-8-50

**Published:** 2007-07-31

**Authors:** Michelle W Chu, Kimberly D Siegmund, Carrie L Eckstam, Jung Yeon Kim, Allen S Yang, Gary C Kanel, Simon Tavaré, Darryl Shibata

**Affiliations:** 1 Department of Pathology, University of Southern California Keck School of Medicine, Los Angeles, CA 90033, USA; 2 Department of Preventive Medicine, University of Southern California Keck School of Medicine, Los Angeles, CA 90033, USA; 3 Department of Medicine, University of Southern California Keck School of Medicine, Los Angeles, CA 90033, USA; 4 Department of Biological Sciences, University of Southern California, Los Angeles, CA 90089, USA; 5 Department of Oncology, University of Cambridge, Cambridge, UK; 6 Department of Pathology, Inje University Sanggye-Paik Hospital, Sanggye 7 dong 761-7, Nowon-gu, Seoul, Korea

## Abstract

**Background:**

Cell division occurs during normal human development and aging. Despite the likely importance of cell division to human pathology, it has been difficult to infer somatic cell mitotic ages (total numbers of divisions since the zygote) because direct counting of lifetime numbers of divisions is currently impractical. Here we attempt to infer relative mitotic ages with a molecular clock hypothesis. Somatic genomes may record their mitotic ages because greater numbers of replication errors should accumulate after greater numbers of divisions. Mitotic ages will vary between cell types if they divide at different times and rates.

**Methods:**

Age-related increases in DNA methylation at specific CpG sites (termed "epigenetic molecular clocks") have been previously observed in mitotic human epithelium like the intestines and endometrium. These CpG rich sequences or "tags" start unmethylated and potentially changes in methylation during development and aging represent replication errors. To help distinguish between mitotic versus time-associated changes, DNA methylation tag patterns at 8–20 CpGs within three different genes, two on autosomes and one on the X-chromosome were measured by bisulfite sequencing from heart, brain, kidney and liver of autopsies from 21 individuals of different ages.

**Results:**

Levels of DNA methylation were significantly greater in adult compared to fetal or newborn tissues for two of the three examined tags. Consistent with the relative absence of cell division in these adult tissues, there were no significant increases in tag methylation after infancy.

**Conclusion:**

Many somatic methylation changes at certain CpG rich regions or tags appear to represent replication errors because this methylation increases with chronological age in mitotic epithelium but not in non-mitotic organs. Tag methylation accumulates differently in different tissues, consistent with their expected genealogies and mitotic ages. Although further studies are necessary, these results suggest numbers of divisions and ancestry are at least partially recorded by epigenetic replication errors within somatic cell genomes.

## Background

Human "age" can be characterized by chronological age (time since the zygote) and mitotic age (total number of divisions since the zygote). Somatic cells within an individual have similar chronological ages but their mitotic ages may differ because different cell types divide at different rates and times. Cell division occurs during normal human development and aging, and many diseases are associated with excessive numbers of divisions. Despite the likely importance of cell division to human pathology, it has been difficult to measure mitotic ages because human lifetimes are long and direct observations or experimental manipulations are impractical.

**Figure 1:**
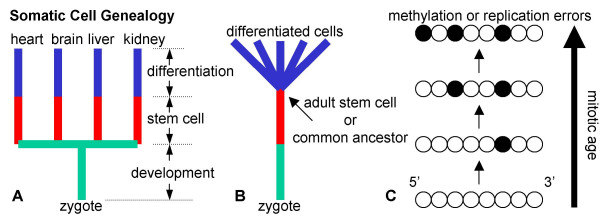
**A human somatic cell tree**. **A**: Every cell has a genealogy that starts with the zygote and ends with its present day phenotype. The genealogy of a differentiated cell can be divided into three phenotypic phases – development from the zygote, a stem cell phase, and differentiation. **B**: Stem cells are common ancestors in a somatic cell tree. The zygote is the ultimate common ancestor, and adult stem cells are more recent common ancestors of differentiated cells. **C**: Replication errors record cell division or genome duplication. Illustrated are a 5' to 3' series of eight CpG sites that are initially unmethylated (open circles). Random replication errors may accumulate during cell division such that some CpG sites become methylated (filled circles). The greater the numbers of divisions since the zygote (mitotic age), the greater the number of errors or methylation (molecular clock hypothesis).

Here we attempt to infer relative somatic cell mitotic ages with a molecular clock approach [1]. Divisions should be surreptitiously recorded within a genome because the greater the number of divisions, the greater the number of replication errors. Molecular clock approaches are commonly employed to study species evolution and infer ancestral trees. All life is thought to be related to a universal ancestor that existed billions of years ago [2]. Sequences differ between individuals and species because genomes cannot be duplicated exactly, and replication errors accumulate. Similarly, all cells within an individual are related, share an ancestral genome, and eventually trace their ancestry back to a universal common ancestor called the zygote (Figure 1). Somatic replication errors are likely to accumulate as genomes are copied during development and aging. Sequence comparisons could be used to infer the mitotic ages of human cells, but sequences mutate too infrequently during a lifetime to effectively function as somatic molecular clocks. For example, point mutations are rare (< 1 per 100,000 bases) even in cancer cell lines [3]. With this error frequency, one would have to sequence millions of bases to identify a few somatic replication errors in normal tissues. Microsatellite loci mutate at higher frequencies and various investigators have proposed or provided data illustrating their potential to reconstruct somatic cell ancestry [4,5].

It may be possible to replace the 5' to 3' order of bases with the 5' to 3' order of CpG DNA methylation because genome replication also involves the duplication of epigenetic patterns. Methylation also exhibits somatic inheritance [6], but is a binary (methylated or unmethylated) code. Unlike sequences, methylation measurably changes with age in certain mitotic human tissues at specific CpG sites [7], suggesting epigenetic replication error rates may be greater than genetic replication error rates.

DNA methylation patterns may be directly compared between different aged individuals because methylation is removed early in development (before implantation) at most CpG islands [8,9]. This early active and passive demethylation essentially synchronizes epigenomes between individuals and potentially an age-related increase in subsequent methylation at certain CpG rich regions ("epigenetic molecular clocks") may represent an accumulation of epigenetic replication errors. Bisulfite sequencing studies of mitotically active epithelium like human endometrium, colon, and small intestines reveal age-related methylation consistent with a "clock-like" accumulation of stochastic replication errors [10-13].

Replication errors may represent the major source of age-related methylation because genomes are regularly duplicated in mitotic tissues. In addition, cell division appears to be required for in vitro de novo methylation [14]. Potentially the mitotic history of any cell can be reconstructed from its methylation patterns if such patterns are not "created" but instead represent a lifelong accumulation of replication errors. Cells with greater mitotic ages would generally have more errors or methylation relative to cells with fewer divisions. However, some methylation may occur independent of division, which would confound a somatic cell clock analysis. To further test whether methylation at "epigenetic molecular clocks" occurs independent of division, brain, heart, kidney, and liver tissues from individuals of different ages were examined. Cell division is minimal in adult brain and heart cells [15,16], and age-related methylation should be absent in these tissues if the majority of tag methylation represents replication errors. Adult liver and kidney cells normally divide infrequently but can divide in response to damage [17,18].

## Methods

### Specimens

Specimens were obtained from 21 autopsies performed at the Los Angeles County-University of Southern California Medical Center (Table 1). The ethical use of the human tissues for research was approved by our Institutional Review Board. Intervals between death and tissue collection were generally less than three days. Heart specimens from 18 individuals were obtained from grossly normal right or left ventricle. Brain specimens from 18 individuals were obtained from grossly normal frontal cortex (primarily gray matter) or cerebellum. Liver specimens from 12 individuals were grossly normal except for the cirrhotic livers from Patients M and Q. Kidney specimens from 18 individuals were obtained from the cortex and appeared grossly normal.

**Table 1:** Specimens

Patient	Age	Sex	Cause of death	Brain	Heart	Kidney	Liver
A	20 wks	M	Stillborn	X	X	X	X
B	22 wks	M	Hydrops fetalis (parvovirus)	X	X	X	
C	32 wks	F	Congenital syphilis	X	X	X	X
D	0 yrs	M	Stillborn, nuchal Cord	X	X	X	X
E	5 yrs	M	Inborn error of metabolism	X	X	X	X
F	25 yrs	F	Cerebral aneurysm	X	X	X	
G	31 yrs	M	Testicular cancer, bleomycin toxicity			X	X
H	33 yrs	M	Cholangiocarcinoma	X	X	X*	
I	36 yrs	F	Cardiomyopathy				X
J	39 yrs	M	Alcoholic cirrhosis	X	X	X	
K	41 yrs	M	Blunt force trauma	X	X	X	
L	48 yrs	M	AIDS	X	X	X	X
M	52 yrs	M	Alcoholic cirrhosis	X	X	X*	X**
N	53 yrs	M	Lung cancer			X	
O	54 yrs	F	Myocardial infarction	X	X		X
P	58 yrs	F	Breast cancer	X	X		
Q	62 yrs	M	Systemic amyloidosis/cirrhosis	X	X	X*	X**
R	70 yrs	M	Sepsis	X	X	X*	X
S	72 yrs	M	Systemic amyloidosis	X	X	X	X
T	81 yrs	M	Diabetes type II	X	X	X*	X
U	84 yrs	F	Gastric cancer	X	X	X*	

* benign nephrosclerosis present

** cirrhosis present

DNA was purified from approximately 25 to 50 mg of tissue using a column method (Dneasy, Qiagen, Valencia, CA). Approximately 500 ng of DNA was bisulfite treated for 12 hours at 50 C using an agarose bead method [11]. Bead sizes were about 25 to 30 ul.

### Methylation analysis

**Figure 2:**
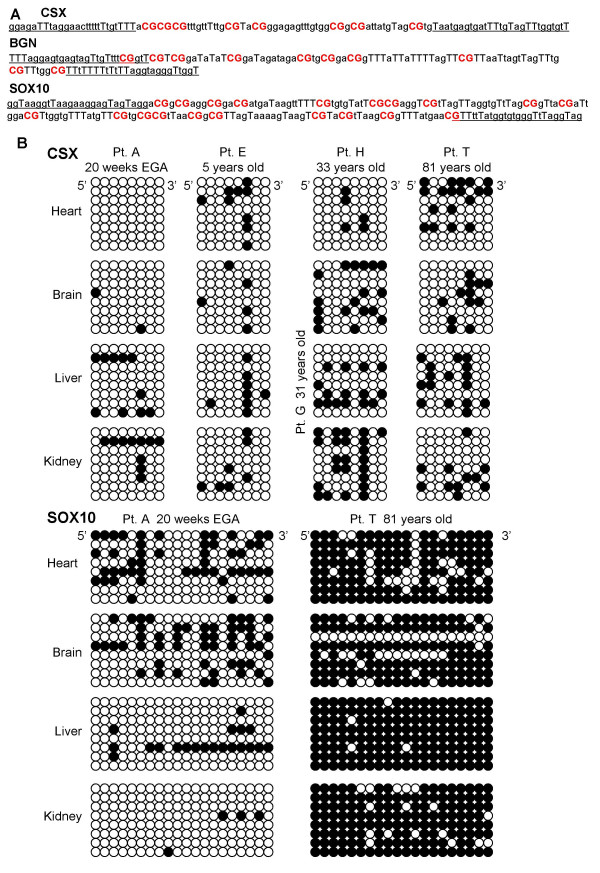
**Epigenetic molecular clocks or tags**. **A**: Sequences (after bisulfite conversion) of the CSX (8 CpG sites), BGN (9 CpG sites), and SOX10 (20 CpG sites) tags. Primer sites are underlined, CpG sites are in bold, and a bisulfite converted "C" at a non-CpG site is indicated by "T". **B**: Examples of CSX and SOX10 tags sampled from the heart, brain, liver or kidney of different aged individuals. The tags are arranged in a 5' to 3' horizontal order with methylated sites indicated by filled circles. There are eight alleles per tissue. Patient H did not have a liver specimen and the liver is instead from Patient G, a 31 year old.

Approximately 10% of the agarose bead containing bisulfite treated DNA was amplified for each PCR (42 cycles) of three different CpG rich loci or tags (Figure 2 and Table 2) that previously exhibited age-related methylation in epithelial tissue [11,19]. The first tag is in the 3' transcribed but untranslated region of the CSX gene (also called NKX2-5). This 51 bp tag contains 8 CpG sites and exhibits age related methylation in epithelial tissues such as the colon, small intestines, and endometrium [11-13]. The second tag is a 151 bp region in exon 2 of SOX10 that contains 20 CpG sites. The SOX10 tag appears to be a relatively fast somatic cell molecular clock because it becomes nearly fully methylated in hair by two years of age [19]. Finally, a 77 bp region in exon 1 of BGN just 3' to the promoter that contains 9 CpG sites and exhibits age related methylation in the colon [11] was examined in the heart and brain of males. The BGN tag is on the X-chromosome, and there is only a single allele per cell in males. PCR products were visualized on an agarose gel and cloned (TOPO TA Cloning kit, Invitrogen, Carlsbad, CA). Individual bacterial clones were sequenced to obtain seven or eight tags per locus per specimen. Clones with evidence of incomplete bisulfite conversion (C's at non-CpG sites) were discarded from the analysis.

**Table 2:** Expression of tag loci*

Gene	Chromosome	Location	Heart	Brain	Liver	Kidney	Colon	Uterus
CSX	5q34	last intron	356**	0	4	0	4	0
SOX10	22q13.1	exon 2	0	0	0	0	0	4
BGN	Xq28	exon 1	401	246	85	653	183	740

* data from website [29], search date 3/5/2007

** transcripts per million

Percent methylation is calculated by dividing numbers of methylated CpG sites by total numbers of examined CpG sites. Statistical analysis used linear regression to test for age-related changes in methylation, and a two-sided t-test for differences between specimen groups. Significance was set at p < 0.05.

## Results

The specimens examined in this study are described in Table 1. Methylation was examined at three CpG rich regions or tags (Figure 2 and Table 2). These tags were initially chosen because they exhibit age-related methylation in epithelial tissues and their genes are not expressed at high levels in epithelium. However, some of the tags are expressed at higher levels in the tissues examined in this study (Table 2). CSX is highly expressed in the heart [20] and BGN has higher expression in the brain and heart. Expression may prevent methylation regardless of cell division because promoter methylation is associated with gene silencing [21]. Of note, only the BGN tag is located close to its promoter in exon 1.

### Mitotic age and stochastic replication errors

Mitotic age cannot be directly inferred from tag methylation because of the random or stochastic nature of replication errors. For example, methylation replication error rates for the CSX tag have been estimated at about 10^-5 ^to 10^-4 ^per CpG site per division [11]. Therefore, many cells will lack tag methylation even though they have divided many times. Cells with tag methylation may have either fewer, the same, or greater numbers of divisions than cells with less tag methylation.

**Figure 3:**
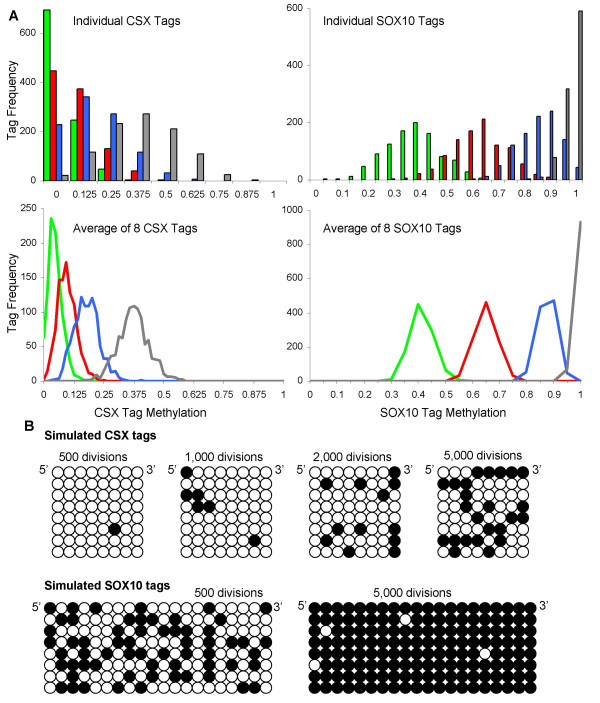
**Simulations of random methylation errors at the CSX and SOX10 tags**. For CSX tags, the simulated error rates are 1 × 10^-4 ^per CpG site per division for methylation and 2 × 10^-5 ^for demethylation. SOX10 tags appear to become methylated faster in human tissues, and the simulated error rate for methylation is greater (1 × 10^-3^) with the same demethylation error rate as CSX. Errors are assumed to be independent between CpG sites, and each tag is an independent genome (no shared ancestry). These simulations are presented to illustrate the general stochastic nature of mutation, and are not an attempt to model the experimental data. **A**: Frequencies of individual tag methylation values and averages of 8 tags after 500 (green), 1,000 (red), 2,000 (blue), or 5,000 (gray) simulated divisions. As expected, values are more scattered for individual versus average tag values. **B**: Examples of simulated individual CSX or SOX10 tags after different numbers of divisions.

The inability to link methylation levels directly with numbers of cell divisions is illustrated by simulation for CSX and SOX10 tags (Figure 3). For a given mitotic age, individual tag methylation patterns and values are scattered. However, average values of eight tags are less scattered. Therefore, the experimental studies are based on average measurements of seven or eight tags per specimen. Consistent with stochastic errors, tissue methylation patterns were diverse (Figure 2).

### Heart

**Figure 4:**
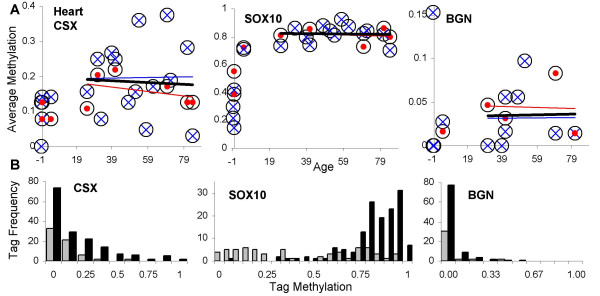
**Heart tag methylation**. **A**: Tag methylation with chronological aging. Circles are averages of eight tags with red solid dots from the right heart, and blue X's from the left heart. Trend lines (black, all values; red, right heart; blue, left heart) were not significantly different from a level line (linear regression, p values > 0.05). **B**: Histograms of individual tags comparing individuals before heart growth is largely finished (≤ 5 years of age, gray bars, N = 64, 63, and 37 alleles for CSX, SOX10, and BGN tags) and adults (black bars, N = 161, 143, and 94 alleles for CSX, SOX10, and BGN tags). CSX or SOX10 tag methylation values were significantly higher in adults.

The heart continues to grow in size after birth, at rates comparable to body weight [22]. CSX and SOX10 tag methylation was significantly lower in fetal, infant and children (≤ 5 years old) hearts and greater in adult (> 21 years old) hearts (Figure 4 and Table 3). Age-related changes in CSX or SOX10 methylation were not observed in adult hearts. BGN heart tag methylation also did not exhibit age-related changes and was generally low (< 5%), with no significant differences between adult and young (≤ 5 years old) hearts. There were no significant differences in tag methylation between the right and left heart.

**Table 3:** Heart and brain tag methylation

Tissue	CSX methylation			SOX10 methylation			BGN methylation		
	≤ 5 yrs old	> 5 yrs old	p*	≤ 5 yrs old	> 5 yrs old	p	≤ 5 yrs old	> 5 yrs old	p
Heart	9.6% (64)**	18.3% (161)	< 0.001	43.4% (63)	81.7% (143)	< 0.001	4.2% (37)	3.6% (94)	0.77
Right heart		15.9% (48)	0.36		81.1% (40)	0.79		4.4% (31)	0.56
Left heart		19.4% (113)			81.9% (103)			3.2% (63)	
	<			<			<		
	≤ 2 yrs old	> 2 yrs old		≤ 2 yrs old	> 2 yrs old		≤ 2 yrs old	> 2 yrs old	
Brain	3.8% (56)	17.3% (160)	< 0.001	44.4% (56)	68.1% (160)	< 0.001	5.6% (40)	8.3% (104)	0.34
Cortex		14.1% (112)	0.005		61.6% (112)	< 0.001		8.7% (72)	0.71
Cerebellum		24.7% (48)			83.1% (48)			7.3% (32)	

* two sided t-test

** numbers of examined tags

### Brain

**Figure 5:**
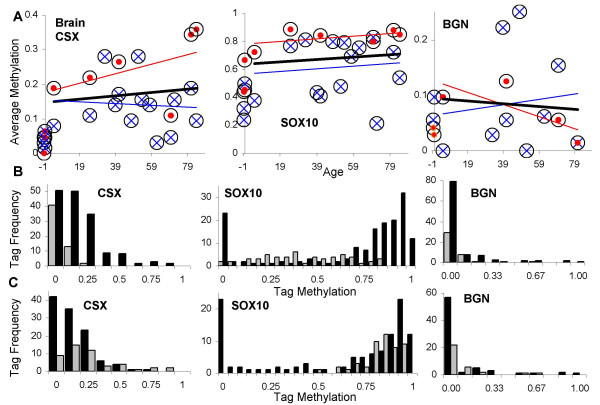
**Brain tag methylation**. **A**: Tag methylation with chronological aging. Circles are averages of eight tags with red dots from the cerebellum and blue X's from the cerebral cortex. Trend lines (black, all values; red, cerebellum; blue, cortex) were not significant different from a level line (linear regression, p values > 0.05). **B**: Histograms of individual tags comparing individuals before brain development is largely finished (< 2 years of age, gray bars, N = 72, 72, and 56 alleles for CSX, SOX10, and BGN tags) and older individuals > 2 years of age (black bars, N = 144, 144, and 88 alleles for CSX, SOX10, and BGN tags). CSX or SOX10 tag methylation values were significantly higher in older individuals. **C**: Histograms of individual tags comparing methylation between the cerebellum (gray bars, N = 40, 40, and 24 alleles for CSX, SOX10, and BGN tags) and the cortex (black bars, N = 104, 104, and 64 alleles for CSX, SOX10, and BGN tags) from older individuals (> 2 years of age). CSX and SOX10 tags were significantly more methylated in the cerebellum. Note that there is a high frequency of completely unmethylated SOX10 tags in the older cortex, which may reflect the presence of neural cells that express SOX10 and therefore preclude methylation (see Discussion).

Brain growth and development occurs early in development and is essentially complete by two years of age [15]. Statistically significant age-related changes in CSX, SOX10, or BGN tag methylation were not observed (Figure 5) after infancy (> 2 years of age). Significantly lower levels of CSX and SOX10 tag methylation were found in neonatal brains (Figure 5B and Table 3). CSX and SOX10 tag methylation levels were significantly greater in the cerebellum compared to the cerebral cortex (Figure 5C and Table 3). However, there was no significant difference in BGN tag methylation between the cerebellum and cortex, or between neonatal and adult brains, but overall tag methylation levels were low (< 10%).

### Liver and kidney

**Figure 6:**
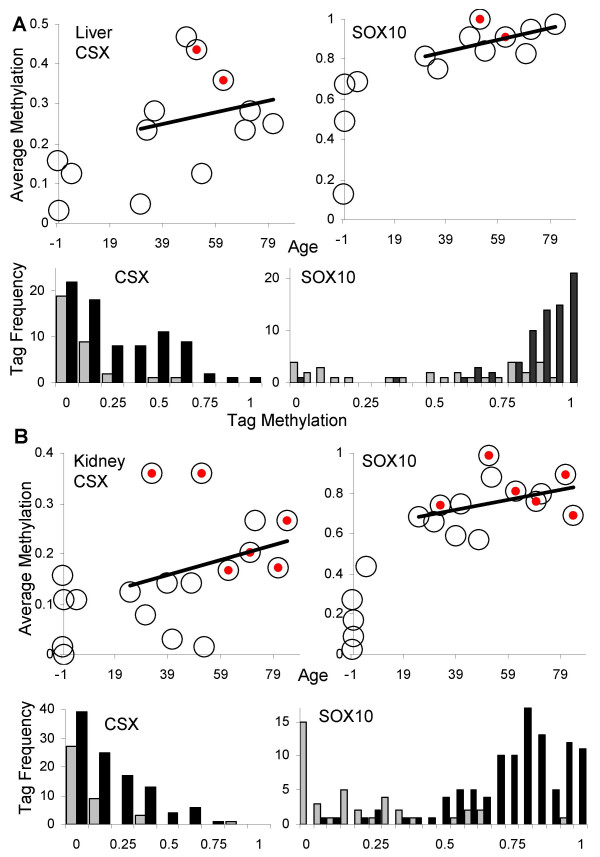
**Liver and kidney methylation**. **A**: Tag methylation with chronological aging in the liver. The trend line indicates an age-related increase in methylation, but the data were not significantly different from a level line (p = 0.38 for CSX, p = 0.19 for SOX10, linear regression). Red solid dots indicate the two samples with cirrhosis. Tag methylation was significantly greater in adults (black bars, N = 80 and 72 alleles for CSX and SOX10 tags) compared to younger individuals ≤ 5 years of age (gray bars, N = 32 alleles for CSX and SOX10 tags). **B**: Tag methylation with chronological aging in the kidney. The trend line indicates an age-related increase in methylation, but the data were not significantly different from a level line (p = 0.59 for CSX, p = 0.097 for SOX10). Red solid dots indicate samples with benign nephrosclerosis. Tag methylation was significantly greater in adults (black bars, N = 105 and 104 alleles for CSX and SOX10 tags) compared to younger individuals ≤ 5 years of age (gray bars, N = 40 alleles for CSX and SOX10 tags).

CSX and SOX10 kidney or liver tag methylation levels were significantly higher in adults relative to individuals less than 21 years of age (Figure 6 and Table 4). There was an age-related increase in tag methylation with adult kidneys or livers, but these trends were not statistically different from a level line. BGN tags were not examined in the kidney or liver.

**Table 4:** Kidney and liver tag methylation

Tissue	CSX methylation			SOX10 methylation		
	< 21 yrs old	> 21 yrs old	p*	< 21 yrs old	> 21 yrs old	p
Kidney	7.8% (40)**	17.9% (105)	0.003	19.9% (40)	75.4% (104)	< 0.001
Liver	8.6% (32)	27.2% (80)	< 0.001	49.4% (32)	88.7% (72)	< 0.001

* two sided t-test

** numbers of examined tags in parentheses.

Cirrhosis was present in two livers, one of which had the highest SOX10 tag methylation and high CSX tag methylation (Pt M). The other cirrhotic liver (Pt Q) had high CSX tag methylation but not remarkably high SOX10 methylation. The liver with the highest CSX tag methylation was from a patient with AIDS (Pt L), with no obvious liver pathology. The two kidneys with high CSX tag methylation had benign nephrosclerosis, which was not observed in kidneys from individuals of similar age with lower CSX methylation. Average CSX and SOX10 tag methylation was higher in kidneys with benign nephrosclerosis, but the average age of individuals with benign nephrosclerosis was also higher, consistent with the observation that nephrosclerosis is more common in the elderly [23].

### Tag changes in multiple tissues

**Figure 7:**
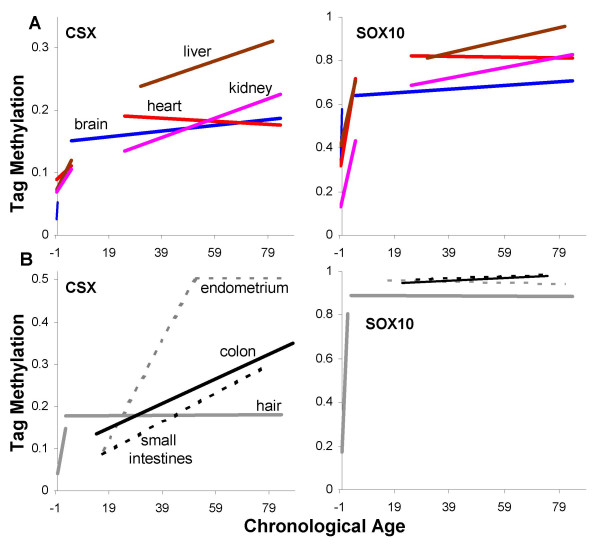
**Average tag methylation with chronological age for multiple human tissues**. **A**: Trend lines for brain (blue), heart (red), liver (brown), and kidney (purple). Negative ages represent fetal tissues (birth equal zero years). **B**: Trend lines for colon (black, data from ref. 11 and additional unpublished specimens), small intestines (black dotted, data from ref. 12), endometrium (gray dotted, data from ref. 13), and hair follicles (gray, data from ref. 19). SOX10 tag values for intestines and endometrium are unpublished data from three different aged individuals (six glands and 48 tags from each organ).

Trend lines for average CSX or SOX10 tag methylation with age for heart, brain, liver and kidney are illustrated in Figure 7A. For comparison with prior studies [11-13,19], average CSX and SOX10 tag methylation with age are also illustrated for colon, small intestine, endometrium and hair in Figure 7B. In general, tag methylation is low around birth and higher in adults. Statistically significant age-related increases in CSX tag methylation were observed in colon, small intestines, and endometrium. SOX10 tags become methylated faster than CSX tags and age-related changes are not observed because they are nearly fully methylated in adult tissues, especially in mitotic epithelium.

## Discussion

Although much is known about human development and aging, the exact fates of somatic cells are somewhat mysterious because it is difficult to observe cells throughout a lifetime. Potentially somatic cell mitotic ages may be inferred by counting numbers of replication errors using a molecular clock approach [10]. Here we provide further evidence that somatic genomes likely record divisions as they are duplicated and physically passed from generation to generation. Certain methylation patterns at specific CpG rich sequences or tags appear to represent somatic replication errors and therefore surreptitiously record mitotic ages.

Prior studies of mitotic adult tissues (intestines and endometrium) demonstrated age-related tag methylation consistent with methylation representing replication errors [11-13]. Cell division is initially required for normal heart and brain development but is infrequent in adulthood [15,16]. Consistent with tag methylation representing replication errors, adult brain and heart tags were methylated but failed to demonstrate age-related increases. The adult tag methylation likely represents replication errors acquired much earlier in life during development because methylation was lower in fetal and infant tissues. Therefore, tag methylation patterns appear to be stable for decades in the absence of division.

Cell division occurs rarely in normal adult liver, perhaps once a year [17]. Consistent with low division rates, tag methylation increased with chronological age, but this trend was not significantly different from a level line. Relatively higher tag methylation levels were observed in livers with cirrhosis, consistent with observations that cirrhosis is associated with hepatocyte proliferation [24]. Division in normal kidney is thought to occur but is relatively rare [18]. Consistent with low mitotic activity, tag methylation apparently increased with age but this trend was not statistically significant. Relatively higher tag methylation was observed in kidneys with benign nephrosclerosis, a relatively common finding at autopsy that increases with age [23].

### Comparisons between different human tissues

A molecular clock approach can compare genomes in cells of widely different phenotype. For example, sequences potentially infer ancestry across all three kingdoms of life [2]. Similarly, a somatic cell clock analysis may allow relative comparisons between cell types if their replication error rates are similar. CSX and SOX10 tag methylation with chronological age for multiple tissues are illustrated in Figure 7. Tag methylation was lower early in life for all tissues, consistent with a start with unmethylated tags and a common origin for their genomes. Methylation or errors increased early in life, consistent with the hyperplasia needed for growth and differentiation. Division largely ceases after birth for the brain or heart, and their mitotic ages and numbers of replication errors appear to remain fixed for a lifetime. In contrast to the heart, brain, liver and kidney, there were statistically significant age-related increases in methylation in the epithelial tissues [11-13]. Generally, endometrium from older women appears to have the highest average mitotic ages, with relatively lower mitotic ages in the heart and brain.

Different tags may have different replication error rates and attain different levels of methylation. Tags with higher error rates better document early development because the mitotic ages of neonatal and infant tissues are relatively low. For example, SOX10 tags become nearly fully methylated by infancy and therefore only minimal changes are possible even with further increases in mitotic age. By contrast, tags such as CSX with lower error rates exhibit fewer changes early in life but more readily document further mitotic age changes during aging.

### Human somatic cell genealogy

**Figure 8:**
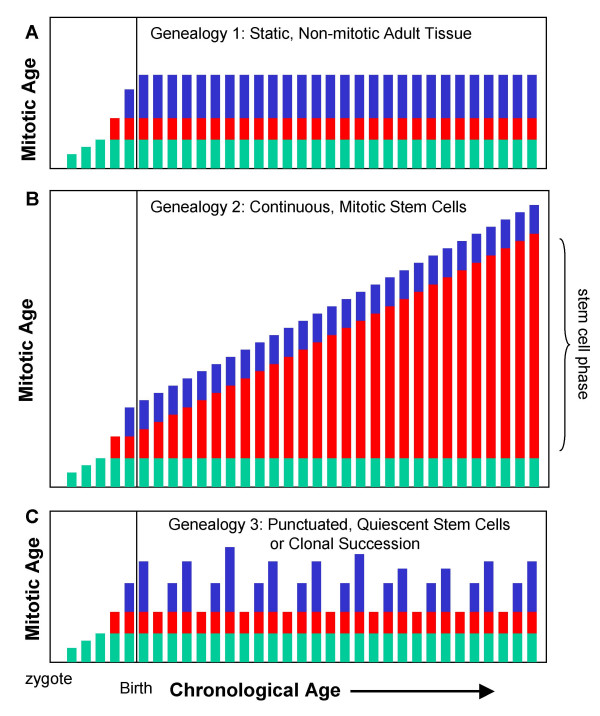
**Types of observed human somatic cell genealogies**. **A**: Static genealogy observed in adult non-mitotic tissues (heart, brain, kidney, liver). Genealogical phases are indicated by green (development from the zygote to the tissue stem cell), red (stem cell phase), and blue (differentiation). Adult genealogies and mitotic ages are fixed with respect to chronological age and replication errors in present day cells accumulated much earlier in life. **B**: Continuous genealogy observed in mitotic epithelium. Only the stem cell phase can vary with chronological age because numbers of divisions are relatively constant during development and differentiation. The mitotic age of a differentiated cell is the mitotic age of its stem cell plus the few additional divisions required for differentiation. An age-related increase in replication errors and mitotic age depends on stem cell divisions. **C**: Punctuated genealogy observed in hair follicles. Average mitotic age and replication errors do not increase with age despite the high hair follicle mitotic activity because hairs and their follicles are cyclically lost and destroyed every few years. Bulge stem cells initiate each new hair cycle, and the mitotic age of a differentiated follicle cell is the age of the stem cell that initiates the new cycle plus the divisions in the follicle. New hairs would have essentially the same mitotic age regardless of chronological age because bulge stem cells are relatively quiescent and only divide a few times at the start of each new hair cycle [26], or if there is clonal succession [25] in which new hair cycles are initiated by previously latent stem cells.

Mitotic age contains additional information about somatic cell genealogy because only certain cell types divide during the differentiation from a zygote to present day cells. Somatic cell genealogy can be divided into three successive phenotypic phases – development from the zygote, a stem cell phase, and differentiation (Figure 1). Genealogy may be confined to certain patterns because numbers of divisions during development and differentiation may be restricted to characteristic numbers and times. We have observed methylation patterns consistent with three basic human genealogies (Figure 8).

The first pattern, observed in non-mitotic adult tissues such as the brain and heart, is a static genealogy. Mitotic age remains constant during chronological aging, and replication errors found in adult cells primarily represent divisions during earlier organ developmental and differentiation. Liver and kidney also have this general pattern, but further stem or differentiated cell divisions may occur during adult life from normal cell turnover or in response to cell loss.

A second pattern, observed in mitotic epithelium such as the intestines and endometrium, is a continuous genealogy that expands as replication errors accumulate throughout life. Replication errors likely accumulate primarily in adult stem cells because stem cell lineages are long lived and numbers of divisions during development and differentiation are usually similar regardless of chronological age. For example, intestinal development is limited to the neonatal period and differentiated cells in the intestines divide only a few times and survive about a week [25]. Therefore, only the stem cell phase can have variable numbers of divisions with aging. The mitotic age of a differentiated intestinal cell is the mitotic age of its stem cells plus the few divisions required for differentiation. Stem cells in the colon, small intestines, and endometrium appear to divide throughout life because their differentiated cells contain greater numbers of replication errors or methylation with increasing chronological age.

A third pattern, observed in hair follicles, is a punctuated genealogy. There is a lack of a measurable age-related increase in average replication errors despite high hair follicle mitotic activity [19]. This pattern may occur if stem cells exhibit mitotic latency, either by dividing infrequently or through clonal succession [26]. Human hair grows for several years and then is lost when the differentiated cells in the mitotic compartment or bulb physically disappears. A new follicle and hair regrow after several months, initiated by stem cells that reside in the hair bulge [27]. Bulge hair stem cells are normally quiescent and divide primarily at the start of a new hair cycle. This stem cell quiescence (infrequent division) or clonal succession (latent stem cells successively divide) ensures that the average mitotic age of hair bulb cells does not measurably increase with chronological age because each new hair cycle starts from a "young" stem cell. Mitotic age is reset when differentiated but mitotic bulb cells (and their errors) are eliminated at the end of a hair cycle, and the next new hair has essentially the same genealogy regardless of chronological age. Physical configuration may influence genealogy, with a punctuated genealogy observed with episodic follicle destruction and reformation, and continuous genealogies observed with glands that persist throughout life.

### Epigenetic clocks and selection

Selection can alter error frequencies by favoring or eliminating some types of errors. Methylation can potentially confer selection because promoter methylation is associated with loss of expression, and promoter methylation is another mechanism to silence tumor suppressor genes in cancer [21]. The loci studied (CSX, SOX10, BGN) were initially chosen because these genes were not highly expressed in epithelium and therefore any methylation would likely be neutral. However, some of these genes are expressed at higher levels in the heart and brain (Table 2), and evidence that expression prevents methylation was observed for the BGN tag, with relatively little methylation (< 10%) even in adult brain or heart. By contrast, CSX is highly expressed in the heart [20], but CSX tags appeared to record mitotic age with less methylation early in life and stable (~18%) adult heart methylation. The CSX tag is in the 3' untranslated region of its gene, which may not affect or be affected by its transcription, whereas the BGN tag is just 3' to the promoter in exon 1. The SOX10 gene is expressed in only some neural tissues, mainly those of neural crest origin [28]. Fully unmethylated SOX10 tags were rarely found after birth in any tissue, except in the cerebral cortex (Figure 5), suggesting SOX10 expression in certain neural cells may preclude or prevent methylation in the region (exon 2) of our tag. Although the mechanism responsible for methylation at our tags is uncertain, methylation does appear to be reduced when the tag is located near an active promoter.

Replication errors are stochastic and the methylation of a single allele is relatively uninformative. Our analysis sampled a relatively small number of tags from only a few different loci, but illustrates the potential for "somatic cell clocks" to record mitotic ages. More information can be obtained with more tags from the same "clock" locus or from multiple "clock" loci. Methylation may not be independent between CpG sites and error rates may vary between CpG sites within a tag or between cell types. However, with most replication error mechanisms, average methylation should generally correlate with mitotic age. Another biological source for tag variation is the sampling of heterogeneous cell types. In this study, organ fragments containing uncertain mixtures of different cell types were analyzed. Ideally tags from small homogeneous clones of cells (as with our prior epithelial studies of single glands [11-13]) would be measured because each cell type may have a distinct genealogy.

## Conclusion

The present study provides tentative evidence that somatic cell mitotic ages may be recorded within their genomes by methylation changes at certain CpG rich loci. The exact mechanisms responsible for methylation changes at our "epigenetic molecular clocks" are uncertain, but many changes appear to represent replication errors. Mitotic age in actively dividing adult tissues may largely depend on the behavior of their stem cells because numbers of divisions during development and differentiation are limited. There are a number of challenges to inferring genealogies from replication errors, but such an approach potentially allows for systematic studies of human development and aging without prior experimental interventions.

## Competing interests

The author(s) declare that they have no competing interests.

## Authors' contributions

MWC, CLE, and JYK performed most of the experiments and helped write the manuscript. ASY and GCK supplied specimens. KDS and ST helped with the statistical and quantitative analysis. DS analysed the data and wrote the manuscript.

## Pre-publication history

The pre-publication history for this paper can be accessed here:


